# Case report: Sotrovimab, remdesivir and nirmatrelvir/ritonavir combination as salvage treatment option in two immunocompromised patients hospitalized for COVID-19

**DOI:** 10.3389/fmed.2022.1062450

**Published:** 2023-01-09

**Authors:** Federico Baldi, Chiara Dentone, Malgorzata Mikulska, Daniela Fenoglio, Michele Mirabella, Federica Magnè, Federica Portunato, Tiziana Altosole, Chiara Sepulcri, Daniele Roberto Giacobbe, Chiara Uras, Graziana Scavone, Lucia Taramasso, Andrea Orsi, Giuseppe Cittadini, Gilberto Filaci, Matteo Bassetti

**Affiliations:** ^1^Department of Health Sciences (DISSAL), University of Genoa, Genoa, Italy; ^2^Infectious Diseases Unit, Polyclinic San Martino Hospital (IRCCS), Genoa, Italy; ^3^Department of Internal Medicine, Centre of Excellence for Biomedical Research, University of Genoa, Genoa, Italy; ^4^Biotherapy Unit, Polyclinic San Martino Hospital (IRCCS), Genoa, Italy; ^5^Hygiene Unit, Polyclinic San Martino Hospital (IRCCS), Genoa, Italy; ^6^General Radiology, Polyclinic San Martino Hospital, IRCCS for Oncology and Neuroscience, Genoa, Italy

**Keywords:** salvage therapy, COVID-19, immunocompromised, monoclonal antibodies, sotrovimab, nirmatrelvir/ritonavir, remdesivir

## Abstract

COVID-19 in immunocompromised patients is difficult to treat. SARS-CoV-2 interaction with the host immune system and the role of therapy still remains only partly understood. There are no data regarding the use of monoclonal antibodies and the combination of two antivirals in fighting viral replication and disease progression. We report the cases of two patients, both treated with rituximab for non-Hodgkin lymphoma and granulomatosis with polyangiitis, respectively, and both hospitalized for COVID-19 with positive SARS-CoV-2 RNAemia, who were successfully treated with a salvage combination therapy with sotrovimab, remdesivir and nirmatrelvir/ritonavir.

## Introduction

There have been several advances among the available therapies for coronavirus disease 2019 (COVID-19), with the development of new oral antivirals and monoclonal antibodies (1–13). Immunocompromised patients have an increased mortality linked to SARS-CoV-2 infection compared to the general population, with high hospitalization and mortality rates of 39 and 34%, respectively (14–17). Furthermore, in this population the negative prognosis seems more related to the virologic phase than to the inflammatory phase (16–18), and the virologic phase could be prolonged with the presence of SARS-CoV-2 in the blood months after the beginning of the infection (19). In a study of 20 patients with lymphoma or undergoing hematopoietic stem cell transplantation, viable virus from nasopharyngeal (NP) swabs was detected by cell culture up to 60 days after the onset of infection (20), while in other reports of hematological patients with SARS-CoV-2 infection the shedding of the viable virus was even more prolonged: 59, 70, 119, and 238 days, respectively (19, 21–23).

The progression over time of SARS-CoV-2 infection in the immunocompromised host can be different than in the general population, and antivirals could maintain a therapeutic role even beyond the first 5–10 days from the beginning of symptoms (24–27).

We report two cases of immunocompromised patients hospitalized in our Infectious Diseases (ID) Unit for COVID-19 pneumonia with SARS-CoV-2 positive RNAemia that were successfully treated with a combination therapy including of anti-Spike monoclonal antibodies (sotrovimab) and oral (nirmatrelvir/ritonavir) and intravenous (remdesivir) antivirals. We also performed the immunological analysis of cellular T responses, before and at the end of salvage therapy, when SARS-CoV-2 RNAemia became negative.

## Case description

### Case 1

A 47-year-old man was admitted to our ID unit in February 2022 for fever, dyspnea, and confusion. He had a history of mantle cell non-Hodgkin lymphoma, treated with autologous hematopoietic stem-cell transplantation in July 2019, on maintenance therapy with rituximab every 6 months (last administration of rituximab in December 2021). The patient was vaccinated against SARS-CoV-2 with two doses of mRNA vaccine (the last dose in April 2021). In January 2022 he had mild COVID-19 not requiring hospitalization, with spontaneous resolution of symptoms and with consecutive negative molecular NP swabs. At the beginning of February, he developed fever (maximum 39°C), accompanied after 4 days by the onset of dry cough. This event was interpreted as a bacterial lower respiratory tract infection, and the patient was treated at home with empirical oral antibiotic therapy (no corticosteroids were used), without resolution of symptoms. Due to worsening conditions, with spikes of high fever, sweating, persistence of dry cough, and confusion, he was admitted to our ID unit.

Blood tests were performed at hospital admission (Day 0): white blood cell 2,640/μl (range 4,500–9,800), neutrophils 2.130/μl (range 1,800–7,800), creatinine 1.2 mg/dl (range 0.67–1.17), mild elevation of liver enzymes (ALT/AST 50/61 UI/ml, range 0–40, 0–40, respectively), lactate dehydrogenase 444U/L (range 135–225), ferritin 1,972 mcg/L (range 30–400), C reactive protein 89.4 mg/L (range 0–3), Interleukin-6 46.5 ng/L (normal value, n.v., <3.4).

**FIGURE 1:**
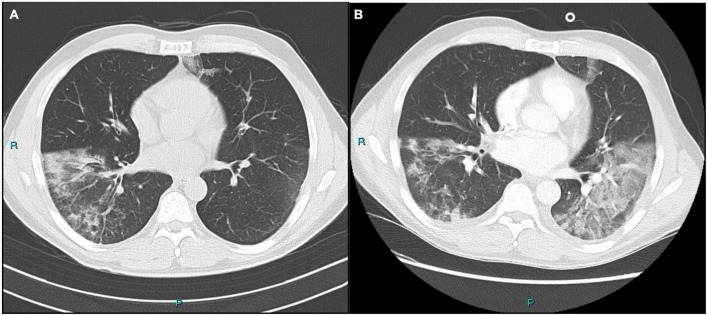
**(A)** Bilateral ground-glass opacities with crazy-paving aspects and more consolidated components, greater at right lower lobe. **(B)** Extension of pulmonary opacities at the left basal lobe compared to the previous imaging.

SARS-CoV-2 serology resulted non-reactive: IgG anti Receptor-Binding Domain (RBD) < 1 U/ml < 12 negative), IgG anti Spike 1 (S1) < 1 U/ml (<21 negative), IgG anti Spike 2 (S2) < 1 U/ml (<9 negative), IgG anti Nucleocapsid < 1 U/ml (<23 negative). Rapid antigenic test and molecular test for SARS-CoV-2 performed on NP swabs were negative on Day 1. Computed tomography (CT) scan performed on Day 1 showed extensive bilateral ground-glass opacities with crazy-paving aspects and more consolidated components, greater at lower lobes in subpleural location (Figure 1A).

Given the radiological evidence of pneumonia in a suspected bacterial infection after the recent SARS-CoV2 infection, a bronchoalveolar lavage (BAL) was performed. Empirical antibiotic therapy with ceftobiprole and levofloxacin was started.

On day 7, due to the worsening clinical conditions, with fever up to 40.5°C and need for supplemental oxygen, he underwent a second CT scan, showing the extension of pulmonary opacities at the left basal lobe compared to the previous imaging (Figure 1B). The antibiotic therapy was changed to ceftolozane/tazobactam plus linezolid.

The same day a RT-PCR on BAL resulted positive for SARS-CoV-2, showing no other concomitant bacterial/fungal/viral infections (culture tests for common bacteria – *Aspergillus spp.* – *M. tuberculosis, M. tuberculosis* DNA*, Pneumocystis jirovecii* DNA, serum β-D-glucan, BAL galactomannan, BAL CMV-DNA, all tested negative). Blood testing for SARS-CoV-2 RNAemia was performed as well on day 7 and resulted positive.

In consideration of worsening bilateral pneumonia in an immunocompromised hematological patient with COVID-19 and no other clear alternative diagnoses for pneumonia, an off-label combination of sotrovimab 500 mg in a single infusion, a seven-day course of IV remdesivir (200 mg of loading dose, 100 mg of maintenance dose) plus a five-day course of oral nirmatrelvir/ritonavir 300 mg/100 mg q12h and intravenous (IV) corticosteroids was then started on day 7. The patient signed the informed consent for off-label treatment.

Sotrovimab was chosen over casirivimab/imdevimab due to the prevalence of variant of concern Omicron BA.1 at that time in Italy, on which the latter has reduced efficacy.

Adjunctive anti-inflammatory treatment (i.e., IL-6 or IL-1 receptor inhibitors) were not deemed necessary considering the presence of SARS-CoV-2 positive RNAemia and the absence of SARS-CoV-2-related inflammatory pattern.

No adverse effects were observed during the course of treatment.

From day 9 the patient remained afebrile, with clinical improvement and consequent reduction in oxygen demand on day 10, until complete weaning to room air on day 13.

On day 12 SARS-CoV-2 RNAemia resulted negative, as well as RT-PCR on NP swab. A follow up PET scan for the non-Hodgkin lymphoma was performed, showing uptake exclusively at the level of the ground-glass pulmonary thickening, suggesting local inflammatory signs of infection and thus ruling out any sign of progression of his hematological disease.

In consideration of the improved conditions and of the reduction of inflammatory parameters, the patient was discharged on day 17.

## Case 2

**FIGURE 2:**
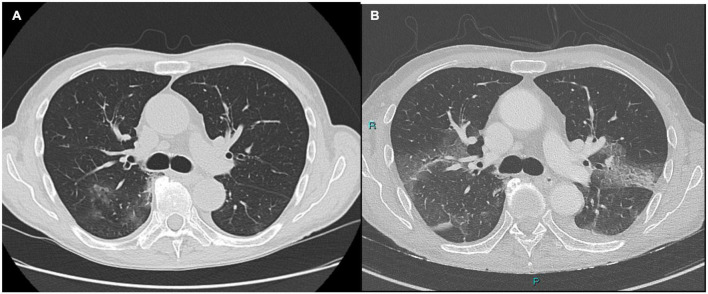
**(A)** Interstitial pneumonia with peripheral small nodules. Postero-basal and para-scissural opacities. **(B)** Ground-glass opacities at the bases of the upper lobes dorsally, with partial periscissural consolidative area. Moderate bilateral pleural effusion with associated disventilatory area.

The second case refers to a 75-year-old man with a history of granulomatosis with polyangiitis with renal involvement on maintenance therapy with rituximab 500 mg every 6 months and prednisone 5 mg q24h (last administration of rituximab in December 2021), a recent finding of atrial fibrillation, and a mitral prolapse. He was vaccinated with three doses of mRNA vaccine (the last in November 2021). He had mild COVID-19 in January 2022, with fever, asthenia and wheezing cough at the onset and a positive SARS-CoV-2 antigenic nasopharyngeal swab, that turned negative 8 days later. Due to the recurrent fever up to 38°C starting at the beginning of February 2022, he underwent a chest CT scan with findings of interstitial pneumonia with peripheral small nodules and ground glass opacities (Figure 2A). He received a 7-day course of oral levofloxacin without resolution of symptoms and was then admitted to our ID unit (day 0). A new SARS-CoV-2 RT-PCR NP swab was performed, which resulted negative.

Blood tests on day 0 showed a normal white blood cell count, D-dimer 428 mcg/L (range 0-500), normal liver function, creatinine 1.0 mg/dL (range 0.67–1.17), hyponatremia (128 mmol/L, range 135–150), C reactive protein 85 mg/L (range 0–3), Interleukin-6 8.8 ng/L (n.v. < 3.4).

SARS-CoV-2 serology resulted non-reactive: IgG anti Receptor-Binding Domain (RBD) < 1 U/ml < 12 negative), IgG anti Spike 1 (S1) < 1 U/ml (<21 negative), IgG anti Spike 2 (S2) < 1 U/ml (<9 negative), IgG anti Nucleocapsid < 1 U/ml (<23 negative).

A new chest CT scan was performed on day 3 and showed extension of the ground-glass opacities at the bases of the upper lobes dorsally, with partial periscissural consolidative area and moderate bilateral pleural effusion with associated disventilatory area (Figure 2B).

To investigate the possible causes of pneumonia, a BAL was requested, however, due to a worsening of the respiratory conditions and hypotension, it was not performed, and an empirical antibiotic therapy with ceftobiprole 500 mg q8gh and methylprednisolone 40 mg q12h was started.

To rule out a possible recrudescence of COVID-19, in the impossibility to perform a BAL, a blood sample was analyzed for SARS-CoV-2 RNAemia and resulted positive (day 6).

An off-label combination therapy of monoclonal antibodies and antivirals was then started on day 6: a single infusion of IV sotrovimab 500 mg, a 7-day course of IV remdesivir (200 mg of loading dose, 100 mg of maintenance dose) plus 5 days of oral nirmatrelvir/ritonavir 300 mg/100 mg q12h.

No adjunctive anti-inflammatory treatment (i.e., IL-6 or IL-1 receptor inhibitors) were deemed necessary due to the presence of SARS-CoV-2 positive viremia and the absence of SARS-CoV-2 – related inflammatory pattern.

No adverse effects were observed.

Five days later, on day 11, SARS-CoV-2 RNAemia resulted negative and on day 12 the patient was weaned off oxygen therapy, with stable peripheral saturation values on room air until discharge from our ID unit on day 14.

In Supplementary Figures 1 and 2 are reported the timelines of clinical conditions, virological tests and different therapies of case 1 and case 2, respectively.

### Immunological analysis before and after salvage combination therapy

For both patients we performed immunological analysis addressing the dynamics of peripheral T cell populations before and at the end of salvage therapy when viremia became negative.

#### Immunofluorescence analyses

Peripheral blood mononuclear cells (PBMC) were purified from heparinized blood samples by centrifugation on Ficoll-Hypaque (Euroclone) gradient. Immunofluorescence analyses were performed on freshly isolated 1 × 10^6^ cells in 100 μl of PBS incubating with specific fluorochrome-conjugated monoclonal antibodies (mAbs) (all reagents for T cell characterization are listed in Table 1 and have been purchased from BD Biosciences). With regards to the analysis strategy to follow the maturation of the CD4+ and CD8+ T populations, T cell maturation has been delineated using a set of canonical markers, i.e., CD45RA, CCR7 as naive (T_NAIVE_), central memory (T_CM_), effector memory (T_EM_), and terminal effector (T_TEM_).

**TABLE 1:** Reagents used for T cell characterization.

Type of marker	Specificity	Clone	Fluorochrome
Cell lineage	CD3	UCHT1	BV786
	CD3	UCHT1	BB700
	CD3	UCHT1	APC-R700
	CD4	RPA-T4	APC-H7
	CD8	RPA-T8	BV421
	CD8	RPA-T8	BB700
	CD8	RPA-T8	PE-Cy7
CD4 + Treg	CD25	M-A251	BV421
	FoxP3	259D/C7	Alexa488
Exhaustion	EOMES	X4-83	PE
	PD-1	NAT105	BV650
	TIM-3	7D3	APC
	CD39	TU66	BV711
Maturation stage	CD45RA	H100	BV605
	CD197 (CCR7)	150503	PE-CF594
	CD197 (CCR7)	150503	PE-Cy7
Effector markers	CD57	NK1	BB515
	GRANZYME	GB11	APC-R700
	Ki-67	B56	FITC
	CXCR5	RF8B2	BV650
Activation markers	HLA-DR	G46-6	PerCP.Cy5, 5
	CD38	HiT2	PE
Dump	Dead cells		LIVE/AQUADEAD

BV, brilliant violet; BB, brilliant bleu; APC, allophycocyanin; PE, phycoerythrin; Cy, cyanine; FITC,fluorescein; PerCP, peridinin-chlorophyll proteins.

#### Multidimensional data reduction analysis

Flow Cytometry Standard (FCS) 3.0 files generated were imported into FlowJo software version 10.7.1 (Becton Dickinson, San Josè, CA), and analyzed by standard gating strategy, and to identify CD3+ CD4+ T cells and CD3+ CD8+ T subpopulations. For each panel data were displayed using t-SNE plot showing median intensity fluorescence of markers with relevant differences between before and after therapies of concatenated samples.

#### Results of immunofluorescence analyses

**FIGURE 3:**
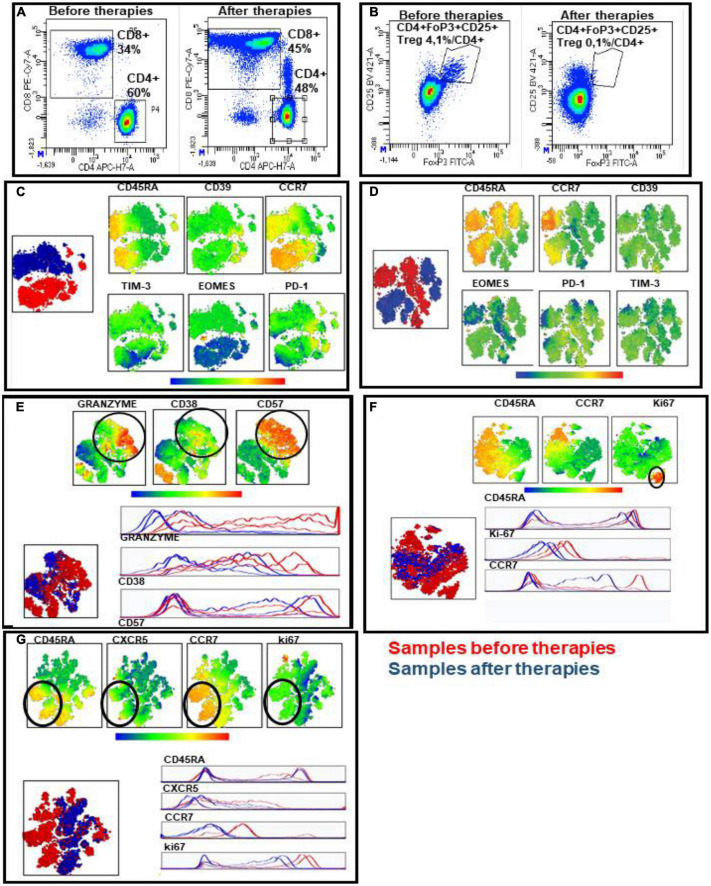
Immunofluorescence analyses of two patients with RT-PCR SARS-CoV-2 positive viremia and bronchoalveolar lavage fluid. **(A,B)** Representative examples of CD4+ and CD8+ T subset percentages on peripheral CD3+ T cells derived from before and after therapies **(A)** and frequency of circulating CD4+ Treg (CD4 + FoxP3 + CD25 +) on circulating CD4+ T population derived from before and after therapies **(B)**. **(C–G)** Multidimensional data reduction analysis. t-SNE analysis of maturation, senescence, exhaustion, effector and activation markers of circulating CD4+ and CD8+ T cells on merged samples derived before (red clusters) and after (bleu clusters) therapies. In particular: the analysis of maturation and exhaustion marker expression in CD4+ **(C)** and CD8+ **(D)** T subsets; the analysis of maturation, senescence and granzyme effector molecule in CD8+ T cells **(E)**; the evaluation of cycling cells by ki-67 marker in CD8+ **(F)** and CD4+ **(G)** T subsets.

The peripheral T cell populations showed that CD4+ and CD8+ T cells clustered differently in samples derived before therapies and after therapies, highlighting profound modifications in both the regulatory and the effector arms of T cell adaptive immunity. Firstly, we observed a decrease in the percentage of circulating CD4+ T lymphocytes and a consequent elevation of the CD8+ counterpart in both patients (Figure 3A) after therapies. As a consequence of this drastic decrease in circulating TCD4+, we also recorded an important reduction in the frequency of circulating CD4 + FoxP3 + CD25 high Treg lymphocytes after therapies (Figure 3B). Multidimensional data reduction analyses (*t*-SNE analysis) performed on peripheral CD4+ and CD8+ T cell populations before and after therapies further highlighted profile expression differences between the two groups of samples derived before therapies and after therapies with regard to maturation, senescence, exhaustion, effector and activation markers. In particular:

1) The analysis of maturation curve and immune checkpoint expression confirmed the different dynamic in samples derived before therapies and after therapies both in CD4+ and CD8+ subsets due to the different viral pressure: (a) to concern the maturative status of CD4+ T lymphocytes, we observed a major cluster of central memory (CM) in samples before therapies, whereas effector memory (EM) and terminal effector memory (TEM) prevail in samples after therapies; consequentially, a major expression of immune checkpoints has been observed in samples after therapies, particularly for transcription factor EOMES analysis (Figure 3C); (b) the *t*-SNE maps of CD8+ T cells relative to the maturation curve also exhibit a progression versus advanced memory cells and a concomitant major expression of immune checkpoints, as EOMES and PD-1 (Figure 3D);
2) The *t*-SNE maps of CD8+ effector population showed that granzyme, relevant effector molecule for cytotoxic activity of T cells, resulted a highly discriminating factor when comparing the samples before and after therapies, in fact, we observed a reduction of granzyme positivity in CD57+ cells, a marker of terminally differentiated cells in non-RNAemic samples; furthermore, the activation level, evaluated by CD38 expression decreased in samples after therapies as expected at the solution of a viral infection (Figure 3E);
3) The evaluation of cycling cells by ki-67 marker shows that: (a) there is a cluster of activated and highly proliferative memory CD8+ T cells (ki-67+, CD45RA-CCR7-) in samples before therapies (Figure 3F); (b) CD4+ T cell comparison also showed a cluster of ki-67+ cells in samples before therapies which exhibits expression of CCR7, CXCR5 markers, showing a presence of T helper follicular (T_FH_) cells only in samples before therapies (Figure 3G). Therefore, the analysis of the CD4+ and CD8+ T cell maps analyzed before and after combination therapy in RNAemic and non-RNAemic samples, respectively, would seem to highlight immunological patterns indicative of a resolution of the infection in the samples derived after therapies, such as the decrease of EOMES expression, the activation status and the reduction of granzyme level.

We analysed the same cellular pattern in another patient with mantle cell lymphoma who underwent treatment with rituximab, bendamustine, cytarabine, with consequent lymphopenia and hypogammaglobulinemia. He was treated in RNAemic phase as antiviral therapy with remdesivir only and steroids and we did not find the same T cellular pattern, but we highlighted in the non-RNAemic phase an increase of granzyme in CD57+ cells and an increase of CD45+ TEM as higher pattern of cytotoxic cells, expression of infection not under control. In fact, that patient developed after few days later a new RNAemic phase of COVID-19 (19).

To confirm if these cellular features observed in CD4+ and CD8+ T cell populations were related to the therapy used, we studied also the circulating CD4+ and CD8+ T cells derived by two other patients (not immunocompromised and not under steroids) treated only with monoclonal antibodies. The *t*-SNE maps showed that CD4+ and CD8+ T cells clustered in a superimposable way in samples derived before therapies and after therapies not showing significantly different patterns in relation to maturation, activation and exhaustion markers (data not shown).

## Discussion

SARS-CoV-2 infection manifests with diverse clinical pictures, suggesting that the severity of symptoms might depend on the outcome of the SARS-CoV-2 immune system interaction within the patient (28–30).

The prolonged shedding of virus and persistent symptomatic SARS-CoV-2 infection have been reported in the immunocompromised patients, mainly in hematological, after B-cell depletion with anti-CD20 antibodies (19, 31–33).

As recently suggested by other experiences, combination therapy with monoclonal antibodies and one antiviral, also for a prolonged time, could contribute to virological success in immunocompromised patients (34, 35).

Sotrovimab and nirmatrelvir/ritonavir are two of the main drugs currently approved for the prevention of severe disease in the outpatient setting, but they are still not approved in the setting of COVID-19 hospitalized patients (36) RECOVERY was the first randomized, controlled, open-label trial to demonstrate the efficacy of the monoclonal antibodies casirivimab/imdevimab (3). Omicron variant showed reduced susceptibility to the use of casirivimab/imdevimab, thus leading to the use of other monoclonal antibodies, such as sotrovimab, in spite of the lack of high certainty evidence for hospitalized COVID-19 patients (37, 38).

During the study period, in our region, the prevalence of Omicron, BA.1 variant, was more than 80%.

Based on the previous assumption and on the therapeutic rationale of applying a combination of different molecules with different mechanisms of action, we chose to adopt an off-label combination therapy of monoclonal antibodies plus two antivirals.

The use of antiviral agents with monoclonal antibodies can have a potential advantage of higher efficacy due to a combination of two different antiviral mechanisms, since antiviral monotherapy might be an insufficient treatment option in the absence of humoral immunity (39). In the case of SARS-CoV-2 infection, *in vitro* data showed synergistic/additive activity of molnupiravir in combination with nirmatrelvir/ritonavir (40). Some recent experiences reported a successful use of combination therapy with antiviral and convalescent plasma or monoclonal antibodies (19, 41).

As of our knowledge, there are no data in literature regarding the use in combination at the same time of sotrovimab, remdesivir and nirmatrelvir/ritonavir, neither *in vitro* nor *in vivo*. Our patients were both treated with this off-label combination with an excellent and rapid clinical response, undetectable SARS-CoV-2 RNAemia and negative RT-PCR SARS-CoV-2 in NP swab.

Concerning the adaptive immune cellular response, all these observations seem to indicate that:

a) The immune system resulted activated and highly responsive to infection in samples before therapies, as demonstrated by the progression to advanced maturational states, positivity for ki-67 marker proliferation and CXCR5 expression in CD4+ T cells significative of active engagement of follicular T helper (T_FH_) cells.
b) After therapies, the dynamic of the clusters of the CD4+ and CD8+ T populations seem to indicate the conditions of an immune system oriented toward the resolution of the infection, as demonstrated by the expression of exhaustion markers, by the reduction of activation levels, absent expression of effector molecules (granzyme) and the decreased proliferative activity.

The humoral or cell-mediated immunity induced by active immunization or response to natural infection, may not develop or develop ineffectively or erratically, thus complicating the assessment of the cause of clinical worsening, thus requiring a different kind of treatment (42, 43). The introduction of new therapeutic options for the treatment of early infection and prevention of progression toward the severe form has further modified the timing of SARS-CoV-2 infection, with the possibility of a late onset of severe clinical manifestations of pneumonia after a few weeks from the onset of symptoms (44).

In our patients, the CD4+ and CD8+ T cells response detected an immunological patterns of a controlled infection as documented in the non-RNAemic samples after therapies, with the decrease of EOMES expression, the activation status and the reduction of granzyme level. These features are different in immunocompromised patient treated with remdesivir alone without control of RNAemic infection phase or in not immunocompromised patients treated only with monoclonal antibodies.

Several studies have now confirmed reduced antibody levels and seroconversion rates in anti-CD20 treated patients following SARS-CoV-2 infection; as B cell could play a role as antigen- presenting cells to naïve T cells, the question remains as whether B-cell depleted patients could still mount functional T cell responses to SARS-CoV-2 (45, 46).

This empiric combination of sotrovimab, remdesivir and nirmatrelvir/ritonavir was safe and apparently effective in our COVID-19 patients with positive RNAemia and a state of immunosuppression, although the inherent limitations of case reports in terms of generalization should be necessary acknowledged. Nonetheless, if confirmed by further investigation, this possible strategy of using a combination of different drugs at the same time could change the approach and the way of how we treat COVID-19 in the immunocompromised patient.

## Data availability statement

The original contributions presented in this study are included in the article/Supplementary material, further inquiries can be directed to the corresponding authors.

## Ethics statement

Ethical review and approval was not required for the study on human participants in accordance with the local legislation and institutional requirements. The patients/participants provided their written informed consent to participate in this study. Written informed consent was obtained from the individual(s) for the publication of any potentially identifiable images or data included in this article.

## Author contributions

FB, CD, MiM, FM, FP, LT, CS, and MaM cared for the patient and supervised diagnostic and therapeutic strategies. DF, CU, GS, and GF supervised and performed immunological analysis. GC provided CT scan and interpretations. AO provided virological analysis. FB and CD wrote the manuscript. DG provided critical review of the manuscript contents and interpretation of data. FB, CD, and MaM performed literature review and provided concepts. MB supervised the analysis and provided concepts. All authors read and approved the final manuscript.
