# Supplementary material for: Case report: Sotrovimab, remdesivir and nirmatrelvir/ritonavir combination as salvage treatment option in two immunocompromised patients hospitalized for COVID-19
Source: Front Med (Lausanne). 2023 Jan 9;9:1062450. doi: 10.3389/fmed.2022.1062450 (PMC9868302; doi:10.3389/fmed.2022.1062450)
Supplement: Supplementary file 1 [file Data_Sheet_1.pdf]

Supplementary figure 1. Timeline of case 1

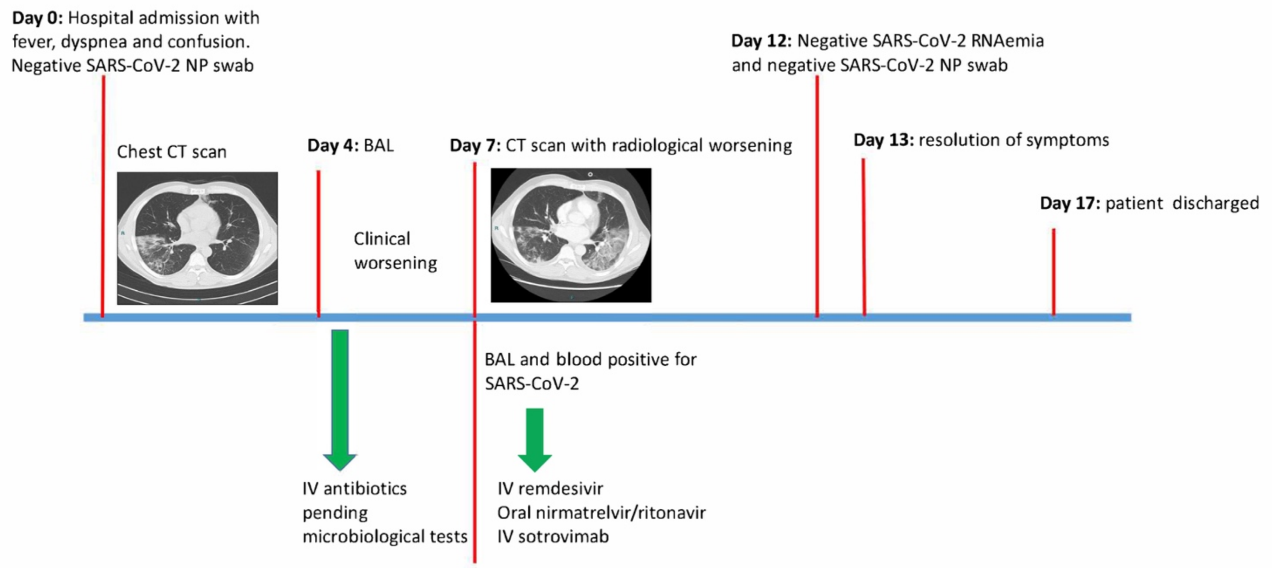

Legend. BAL, bronchoalveolar lavage; CT, computerized tomography; IV, intravenous; NP, nasopharyngeal.

Supplementary figure 2. Timeline of case 2

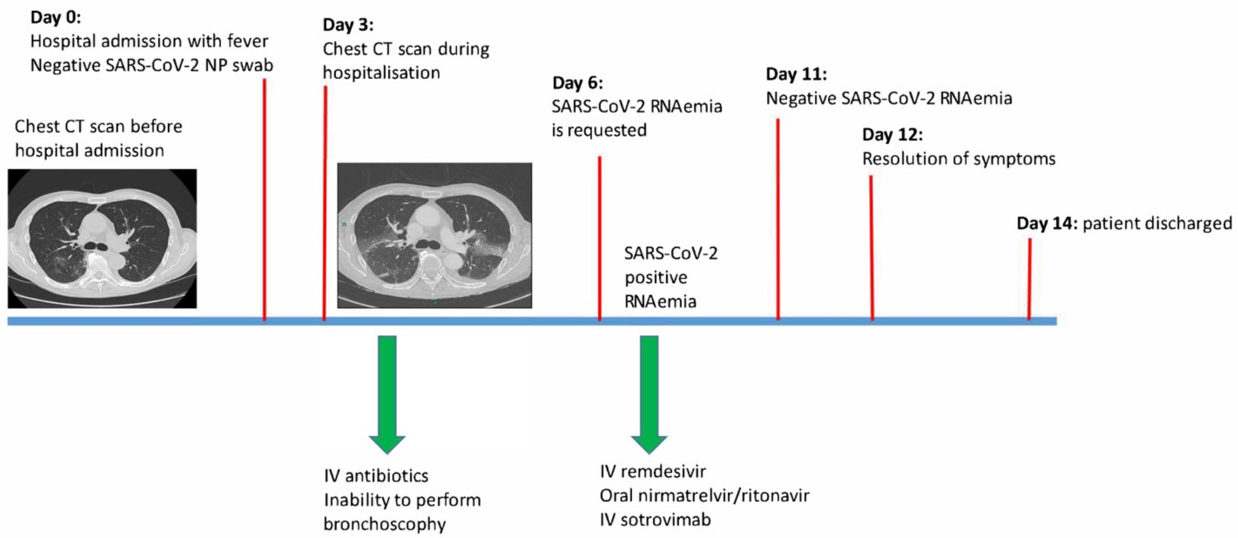

Legend. BAL, bronchoalveolar lavage; CT, computerized tomography; IV, intravenous; NP, nasopharyngeal.
